# Impact of fine arts education on psychological wellbeing of higher education students through moderating role of creativity and self-efficacy

**DOI:** 10.3389/fpsyg.2022.957578

**Published:** 2022-08-09

**Authors:** Xuguang Jin, Yuan Ye

**Affiliations:** ^1^College of Fine Arts, Shaanxi Xueqian Normal University, Xi'an, China; ^2^Xi'an Yuying Primary School, Xi'an, China

**Keywords:** fine arts education, psychological wellbeing, creativity, self-efficacy, China

## Abstract

The purpose of our research was to explore the impact of fine arts education on psychological wellbeing among undergraduate students through moderating role of creativity and self-efficacy. Art is the most effective medium for expressing human ideals, culture, identity, lifestyles, emotions, and societal experiences. Cross-sectional research was carried out on 376 undergraduates in the 2022–2023 academic year at the public and private Chinese universities, and those students who are currently enrolled in fine arts courses. A link to the Google Doc survey was sent through email and social media channels (i.e., WeChat). The time frame of the data collection was 3 months, from February 2022 to April 2022. While analyzing the obtained data, we used IBM SPSS version 25, which includes both descriptive and inferential statistics. The overall results of the study indicate that the fine arts education positively and significantly influences psychological wellbeing. Moreover, findings also indicate that the creativity and self-efficacy positively and significantly moderate the relationship between fine arts education and psychological wellbeing. The study highlighted the significance of fine arts education in Chinese students. Through this study, students studying in this field should be made more aware of the importance of fine arts education and its link with psychological wellbeing. Further, art courses should be added to the curriculum at different levels of education to boost the creativity and self-efficacy of higher education students in China. Implications for parents, students, and teachers are also discussed.

## Introduction

Education is one of the effective processes for individual development. In this regard, art is one of the most important building blocks of developed societies as part of education. Art education is fundamental to the human experience. Since art is a vital part of science and technology, it is the only way to raise people who can see clearly, perceive reality clearly, think analytically, question, break free from rigid conventions, create novel patterns geared toward development, and significantly contribute to the advancement of societies (Yeniasır and Gökbulut, 2018). The success of an educational institution is highly dependent on student motivation, educational fulfillment, and joy at their educational institution (Yafi et al., 2021). To increase the standards of education, it is crucial to maintain a high level of educational quality and assess the educational satisfaction of students (Demirbatir, 2020). Besides, university education causes psychological difficulties for students due to a variety of reasons, including moving to a new city, living apart from their families, and increasing responsibilities for their own decisions. Also, it is possible that factors such as encountering a different environment than the student expected, making the program harder than expected, or not being suitable for him/her may be effective in the student's burnout, vitality, and education satisfaction (Demirbatir, 2020). Despite the strong promotion of aesthetic education to knowledge in China, the subject of visual arts is still neglected by the general public (Yue, 2022).

While the arts have always been difficult to define conceptually, there are a number of cross-cultural characteristics that are recognized as fundamental to art. These include the art object (physical or experiential) being valued in its own right rather than merely as a utility; providing imaginative experiences for both the producer and the audience; and containing or provoking an emotional response. Also, the production of art is distinguished by the need for novelty, creativity, or uniqueness; the need for specialized skills; and adherence to the rules of form, composition, or expression (Fancourt and Finn, 2019).

In terms of health research, engagement with the arts has been classified into five broad categories: performing arts like activities in the genres of music, dance, theater, singing, and film; visual arts, design, and craft such as crafts, design, painting, photography, sculpture, and textiles; literature like writing, reading, and attending literary festivals; culture, including going to museums, galleries, art exhibitions, concerts, the theater, and community events (Davies et al., 2012). Arts activities are complex or multimodal interventions because they combine multiple components that are all known to be health promoting (Craig et al., 2008).

People inhabit a world that is physical, emotional, mental, and spiritual and can acquire cognitive skills from various courses. However, students cannot satisfy their emotional and spiritual demands with classes such as mathematics, chemistry, and foreign languages. In this view, it is possible to claim that art is the most significant source of the sensitivity that influences the development of humans (Mercin and Alaku, 2007). For instance, fine arts education has numerous advantages for students of all ages. Learning through the fine arts in all of their forms, such as dance, music, theater, and visual arts, encourages students to think creatively, imaginatively, and innovatively (Arts Education Partnership, 2016). Likewise, as they participate in learning activities that include the fine arts, students develop instrumental skills such as critical thinking, problem-solving, communication, and collaboration. Prior research studies have found that learning through the fine arts improves academic performance and student engagement in school, social, and community activities (Luftig, 2000; Ingram and Riedel, 2003; Snyder and Cooper, 2015).

The purpose of our research was to explore the impact of fine arts education on psychological wellbeing among higher education Chinese students through moderating role of creativity and self-efficacy. In the education sector, art is one of the most effective media for expressing human ideals, culture, identity, lifestyles, emotions, and societal experiences (Zeb et al., 2021). In the course of history, art was strengthened by humans and, consequently, civilizations, and passed from one person to the next. Therefore, education is the most effective means of shaping, raising, developing, leading, and preparing for the future healthy persons and their communities. This expectation, however, is closely related to the quality of education designed and implemented. Individuals and societies both require artistic and cultural education. Besides, painting, sculpture, architecture, visual communication, photography, cinema, music, dance, theater, literature, design, and other areas of the fine arts are all taught and trained in general educational institutions. University is a time when young people face a variety of issues, including academic, personal, financial, peer pressure, and parental conflicts. Because these issues have an impact on an individual's psychological wellbeing, the role of fine arts education in dealing with them is important. Furthermore, an individual's ability to learn arts-related skills and the role of creativity are important factors. However, few research studies were previously conducted in relation to these variables in China. Therefore, this study was conducted on Chinese students to explore the impact of fine arts education on psychological wellbeing and how the role of creativity and self-efficacy acts as moderators among higher education Chinese students.

## Research literature

### Psychological wellbeing theory

Ryff (1995) developed a theory and model of psychological wellbeing and identified six interconnected but distinct aspects related to the eudaimonic aspect, namely, self-acceptance, positive relationships, environmental mastery, personal growth, autonomy, and life meaning (Snyder and Lopez, 2007). These factors were used in this study because they are the most commonly used measures of positive psychological functioning (Ryff, 1995). According to an existing literature review, psychological wellbeing is a unified theoretical framework. It has long been hypothesized that mental health includes high levels of emotional, psychological, and social wellbeing, as well as the absence of mental illness (Ryff and Keyes, 1995).

### Investment theory of creativity

Sternberg and Lubart (1999) proposed the investment theory of creativity, which states that intellectual abilities, adequate knowledge, the ability to think creatively, personality traits, intrinsic motivation, and a supportive environment are all aspects of creativity. Sternberg and Lubart (1992) distinguished two types of approaches to studying creativity, namely, person-centered approaches and context-centered approaches. Person-centered approaches emphasize internal, contextual aspects of creative performance, whereas context-centered approaches emphasize the individual's interaction with the external context in which he or she lives. Arshad and Rafique (2016) believes that environmental variables interact with cognitive variables to produce creative behavior, and that the availability of materials and resources facilitates creative behavior.

The Four P framework, which focuses on the four Ps, namely, person, product, process, and press, is one of the most important fundamental theoretical frameworks on creativity. Based on the previous theory, the Four C framework was developed in 2007. Beghetto and Kaufman (2007) proposed this theory, distinguishing the little C from the big C, with the former referring to everyday creativity and the latter referring to exceptional creativity (Paul and Elder, 2009). Amabile and Pratt (2016) highlighted the componential model of creativity, which includes three interconnected variables, namely, domain-relevant skills, creativity-related process, and intrinsic and extrinsic motivation. Others have investigated the driving force for people to become creative, in addition to studying the key components of creativity. According to Csikszentmihalyi (1988), one of the most important motivators for creativity is flow, which refers to achieving a sense of fulfillment simply by doing the things one enjoys doing (Hu and Han, 2021).

### Impact of fine arts education on psychological wellbeing

Psychological wellbeing is defined as a state of health, happiness, and prosperity. It entails feeling good and performing well in our daily lives (Elliott and Gramling, 1990). Elements such as positive and negative affect, happiness, life satisfaction, creative thinking, prosocial behavior, and good physical health are all associated with psychological wellbeing (Sheldon and Kasser, 1998; Diener and Biswas-Diener, 2008). For instance, greater psychological wellbeing is linked to a variety of physical and mental health benefits, including a stronger immune system, better sleep patterns, lower blood pressure, and even longer life (Carr, 2004). The concept of wellbeing contains personal, emotional, social, psychological, and spiritual dimensions, as well as health-related behaviors (Snyder and Lopez, 2007). Besides, it is based on two main types of wellbeing, namely, hedonistic (subjective and emotional) and eudaimonic (psychological and social) (Snyder and Lopez, 2006). Subjective wellbeing is another name for hedonistic wellbeing, which focuses more on how people feel and what makes them happy in life. It has both an emotional (high positive affect and low negative affect) and a mental (thinking) part (satisfaction with life). The focus of eudaimonic wellbeing is on the psychological and social parts of human functioning that show and help people work toward important life goals. This is usually called as “psychological wellbeing” (Snyder and Lopez, 2006).

Aesthetic experience involves the perception of aesthetic objects and the pleasure that results. This pleasure is not gained from the goods' practical features, but rather from the intrinsic qualities of the aesthetic objects themselves. Aesthetic experiences can result from the appreciation of attractive human artifacts, such as artworks (e.g., poetry, sculpture, music, visual arts), or aesthetic nature objects, such as sunsets or mountain views (Mastandrea et al., 2019). Recent research indicates that the arts can boost health and psychological wellbeing and serve as a therapeutic aid for many, including teenagers, the elderly, and those who are vulnerable (Daykin et al., 2008; Todd et al., 2017; Thomson et al., 2018). Higher education uses the fine arts as its medium. It educates and cultivates students in the areas of emotions, thinking, morals, values, innovation, practical skills, and aesthetics. It is also for this reason that fine arts education in higher normal institutions should emphasize training students' professional knowledge and abilities in fine arts, and take it seriously in order to improve their artistic and cultural achievements, as well as innovation and practice. In this approach, universities can assist students in enhancing their character and morality, as well as their moral, intellectual, physical, aesthetic, and professional growth (Jing, 2014).

As a starting point in the art education approach, the student should accept the cultures to which he belongs. This is the best possible outlet for establishing the trust that comes from a deep appreciation of one's own culture, as well as for exploring the cultures of others and then respecting and appreciating them. Accepting the constant evolution of culture and its value in both historical and contemporary contexts is central to this. In this context, art should be gradually introduced to students through artistic practices and experiences, while also preserving the value of the process itself. Additionally, as many art forms cannot be confined to a single discipline, more emphasis should be placed on the interdisciplinary aspect of art and its commonalities (UNESCO, 2006). Heise (2004) stated that “the merging of art education and visual culture can provide learning experiences that prepare pupils to participate in a democratic society.” Based on the abovementioned literature, Hypothesis 1 was formulated as follows:

H1: Fine arts education has a positive influence on psychological wellbeing.

### Role of creativity as a moderator between fine arts education and psychological wellbeing

Creative thought is characterized by Goldenberg and Mazursky (2002) as “a process that may be channeled, diagnosed, and reconstructed using analytical procedures.” Sternberg and Lubart (1999) also described creativity as “the development of novel, i.e., original and surprising; appropriate, i.e., valuable things.” To enhance the quality of undergraduate education, students must engage in research and creative endeavors. The value of creativity in learning and accomplishment, according to Jackson et al. (2006), is recognized in the field of higher education, despite the fact that creative aptitude and inventiveness are the greatest human assets and achievements (Hilala et al., 2013).

Creativity plays a role as a moderator between fine arts education and psychological wellbeing among undergraduate Chinese students. Art education, which includes visual arts activities such as drawing, painting, sculpting, designing, and collage, is at best peripheral and must be defended within the regular school curriculum. It is increasingly recognized that the arts contribute significantly to students' academic achievement, and that art education contributes to students' aesthetic development, as well as the development of thinking and creativity. For instance, King and Pope (as cited in Carr, 2004) found a link between creativity and various psychological traits such as autonomy, introversion, and openness to new experiences. Gingantesco et al. (2011) also discovered that some creative people are predisposed to depression and anxiety symptoms, and thus report lower psychological wellbeing. Thus, recent education reform has initiated efforts to promote art education as a part of a balanced education aimed at students' whole-person development, which is essential to youths' intellectual, emotional, and creative growth (Chan and Chan, 2007). Therefore, we developed the following hypothesis:

H2: Creativity plays a moderating role between fine arts education and psychological wellbeing.

### Role of self-efficacy as a moderator between fine arts education and psychological wellbeing

Albert Bandura introduced the concept of self-efficacy into social cognitive theory (1977). The belief of self-efficacy is that an individual can perform the behavior required for a specific outcome, resulting in an increase in an individual's high self-efficacy (Bandura, 1977). Fine arts education is extremely useful in terms of the expected impact on the individual's personality because it allows the individual to realize his/her own abilities, and gain and develop a sense of self-confidence between the individual and social goals. As a result, fine arts education is both necessary and mandatory in terms of learning the art of social rise, application, and intellectual dimension (Romanescu and Özlem, 2020). For example, individuals who have a positive physical assessment of themselves are safer in their interpersonal relationships and more successful in their professions, whereas those who dislike themselves and believe they have many flaws experience uneasy, unsafe, and worthless feelings throughout their lives (Ermis and Imamoglu, 2019).

**Figure 1:**
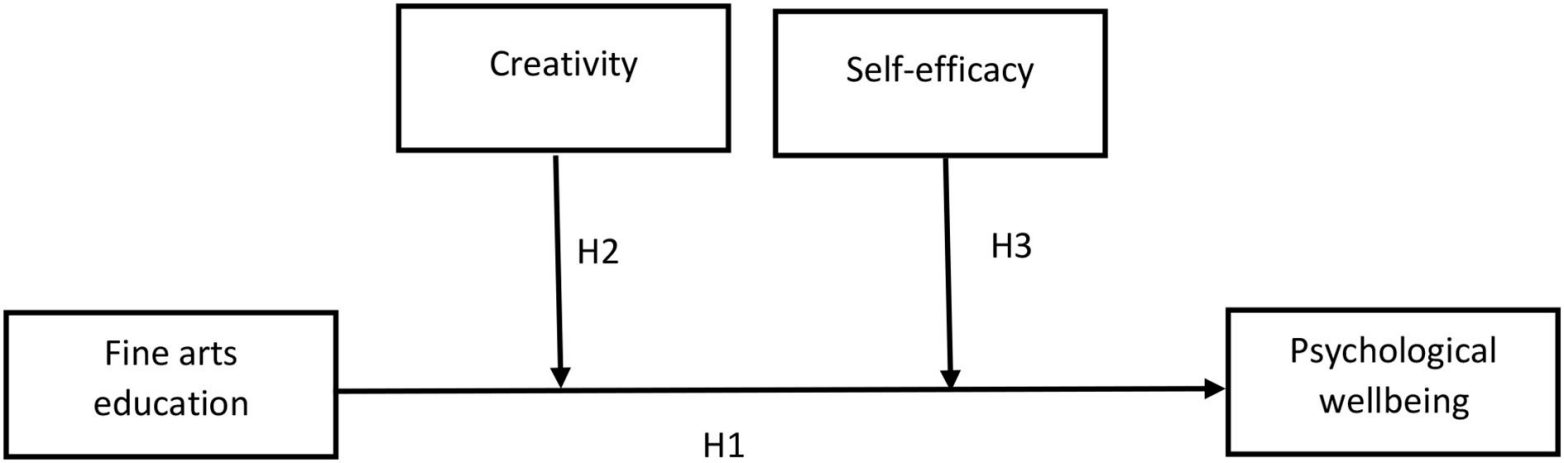
Theoretical model.

Self-efficacy is neither a purpose, a demand for control, a psychological feature, nor a result expectation (Ran et al., 2022). Rather, it is the capacity to integrate desired goals with one's potential, abilities, and skills in specific situations. It plays a major part in a variety of common psychological disorders. Low self-efficacy is directly correlated with avoidant behavior, sadness, and dysfunctional anxiety (Kausar and Ahmad, 2021). Besides, psychological wellbeing represents a person's affiliation with others and self-referent attitudes, i.e., growth-oriented, mastery-oriented individuals. It is related to intra- and inter-individual positive and affective functioning. It illustrates dimensions of affective life satisfaction evaluations. Psychological wellbeing reflects a multidimensional perspective, which is comprised of six unique areas, namely, personal growth, autonomy, life purpose, environmental mastery, positive relatedness with others, and self-acceptance (Burns, 2016). Self-efficacy is associated with a variety of psychological problems. People with depressive mood tendencies believe they are completely incapable and a failure. It is widely acknowledged that performing arts are an extracurricular activity that contributes to the psychological wellbeing of students by fostering increased self-esteem and social skills (Kausar and Ahmad, 2021). Therefore, self-efficacy plays a role as a moderator between fine arts education and psychological wellbeing among undergraduate Chinese students (see Figure 1). Based on the abovementioned literature, we developed the following hypothesis:

H3: Self-efficacy plays a moderating role between fine arts education and psychological wellbeing.

### Research methodology

Cross-sectional research was carried out on undergraduates in the 2022–2023 academic year at the public and private Chinese universities, and those students who are currently enrolled in fine arts courses. A link to the Google Doc survey was sent through email and social media channels (i.e., WeChat). All respondents were educated about anonymity, participation, and withdrawal rules on the first page of the survey. The time frame of the data collection was 3 months, from February 2022 to April 2022. At the end of the data collecting period, 405 replies were collected; however, 29 responses were estimated insufficient, leaving a total of 376 valid responses. The demographics information of the respondents is shown in Table 1. Briefly stated, 49.7% of the respondents were females and 50.3% were males. Between the ages of 17 and 23 years, 78.2% participated in the study, followed by those between the ages of 24 and 29 years with 19.1%. Chinese made up the majority of the participants (64.1%), with Malay, Indian, and other ethnic groups making up the remainder. There were 67.3% of participants from private universities, and only 32.7% from public universities. An aggregate of 450 surveys were sent and 376 responses were received. It was encouraging to see a response rate of 83.55% in such a challenging COVID-19 time period.

**Table 1:** Descriptive statistics.

**Demographics**	**Frequency**	**Percent**	**One-way ANOVA**
Gender			0.381
Female	187	49.7	
Male	189	50.3	
Age			0.001
17–23	294	78.2	
24–29	72	19.1	
30–33	10	2.7	
Ethnicity			0.006
Chinese	241	64.1	
Malay	83	22.1	
Indian	44	11.7	
Others	8	2.1	
University			0.447
Public	123	32.7	
Private	253	67.3	

There were both Chinese and English versions of all the scales used in this research. The independent variable fine arts education was measured using a 19-item scale developed by Maus (1938). The dependent variable psychological wellbeing was adapted from Ryff and Keyes (1995), based on an 18-item scale. Finally, two moderating variables, namely, creativity, based on a 50-item scale developed by Kaufman (2012), and self-efficacy, based on the 50-item scale adapted from Schwarzer and Jerusalem (1995), were used. A five-point Likert scale was used: 1—definitely not; 2—somewhat not; 3—neither yes or no; 4—somewhat yes; and 5—definitely yes. An initial pilot study was undertaken to ensure that the scales used for data collecting were consistent. There were no significant differences in reliability between the Chinese and English versions of the measures used in the pilot research.

## Results

While analyzing the obtained data, we used IBM SPSS version 25 (Morgan et al., 2019), which includes both descriptive and inferential statistics. The analysis was performed in two major stages: Cronbach's alpha, skewness, kurtosis, Kaiser–Meyer–Olkin (KMO), and Bartlett's test of sphericity were applied in the first stage to analyze the gathered data, and in the second stage, a regression analysis was used to examine the impact of fine arts education on students psychological wellbeing through moderating role of creativity and self-efficacy. The reliability of a research instrument is characterized by its accuracy. Cronbach's alpha values range between 0 and 1, and a value of 0.70 or above demonstrates the instrument's reliability for the research under consideration (Bonett and Wright, 2015). As demonstrated in Table 2, the Cronbach's alpha values of all the variables were adequate and over the 0.70 threshold limit. The degree of reliability used to measure a parameter or concept in quantitative research is referred to as the degree of data validity (Bonett and Wright, 2015). The factor loading values were determined to be greater than the minimal threshold value of 0.4 (Field, 2013), indicating that the items were legitimate for this research and no item was needed to be deleted.

**Table 2:** Reliability, validity, and normality analysis.

**Variable**	**Items**	**Skewness**	**Kurtosis**	**α**	**KMO**	**Bartlette's Test of Spehericity**	**References**
Fine arts education	19	−0.463	−0.363	0.892	0.896	1296.05	Maus, 1938
Psychological wellbeing	18	−0.364	−0.742	0.910	0.732	2364.71	Ryff and Keyes, 1995
Creativity	50	−0.306	−0.936	0.923	0.894	1346.65	Kaufman, 2012
Self-efficacy	10	−0.420	−0.486	0.885	0.881	951.01	Schwarzer and Jerusalem, 1995

The analysis of the data reveals that both the skewness and kurtosis values fall within the acceptable ranges, indicating that the data collected for this research are normally distributed. As the values of skewness are between −1 and +1 and those of kurtosis are between −3 and +3, the study's data are acceptable (Groeneveld and Meeden, 1984). In addition, Bartlett's test of sphericity was statistically significant (*p* = 0.000) and the KMO values for all variables were more than 0.5, indicating that the obtained data are useful for further analysis (Williams et al., 2010).

Pearson's correlation coefficient (*R*) was used for correlation analysis, with values ranging between −1 and +1 (Adler and Parmryd, 2010). The negative and positive signs indicate an inverse or direct relationship, respectively. For a strong relationship between latent variables, Pearson's coefficient should be more than 0.5, between 0.3 and 0.5 for a moderate relationship, and <0.2 for a weak relationship. Table 3 shows that the correlation between fine arts education and control variable is not significant with age (*R* = −0.058, *p* > 0.01) and ethnicity (*R* = −0.023, *p* > 0.01), and the correlation between fine arts education and psychological wellbeing (*R* = 0.743, *p* < 0.01), creativity (*R* = 0.860, *p* < 0.01), and self-efficacy (*R* = 0.436, *p* < 0.01) is shown to be significant and positive. Similarly, the correlation between psychological wellbeing and control variable is not significant with age (*R* = −0.046, *p* > 0.01); however, it is negatively significant with ethnicity (*R* = −0.135, *p* > 0.01). Moreover, the correlation between psychological wellbeing and creativity (*R* = 0.747, *p* < 0.01) and self-efficacy (*R* = 0.317, *p* < 0.01) was also positive and significant (Table 3).

**Table 3:** Correlation analysis.

**Variables**	**Age**	**Ethnicity**	**Fine arts education**	**Psychological wellbeing**	**Creativity**	**Self-efficacy**
Age	1					
Ethnicity	0.093	1				
Fine arts education	−0.058	−0.023	1			
Psychological wellbeing	−0.046	−0.135**	0.743**	1		
Creativity	−0.072	−0.025	0.860**	0.747**	1	
Self-efficacy	−0.100	−0.021	0.436**	0.317**	0.464**	1

^**^Correlation is significant at the 0.01 level (two-tailed).

For the purpose of validating the existence of the relationship between the variables, correlation analysis has been employed, which demonstrates that variables are interrelated. However, correlation analysis alone is insufficient because it merely demonstrates the existence of the relationship between the studied variables and does not provide sufficient evidence to clarify the underlying relationship between the variables (Abadie et al., 2020). To test direct effect of fine arts education (FAE) on psychological wellbeing (PW) and indirect effect through moderating variables creativity (CR) and self-efficacy (SE), we used model 2 of PROCESS macros using SPSS version 25 (Hayes, 2017).

Table 4 displays the results of testing hypotheses. First, the hypothesis “Fine arts education has a positive influence on psychological wellbeing” was investigated (see Figure 1). The results demonstrate a positive and statistically significant association between fine arts education and psychological wellbeing (β = 0.3819, *p* < 0.000). The value of β indicates the percentage change, indicating that a one-unit change in fine arts education results in a 0.3819 unit change in psychological wellbeing. The findings suggest that >38.19% of change is detected in psychological wellbeing, and the *p*-value of 0.000 indicates a high degree of significance, providing solid grounds for accepting H1. Furthermore, the moderating effect of creativity (β = 0.3512, *p* < 0.000) and self-efficacy (β = 0.4010, *p* < 0.000) was also positive and significant. The lower limit confidence interval (LLC) and upper limit confidence interval (ULCI) values have no zero between both limits, which clarifies that the results are significant. Hence, H2 and H3 are also accepted.

**Table 4:** Regression analysis.

**Hypotheses**	**Constructs**	**β**	**se**	**T**	**p**	**LLCI**	**ULCI**
	Control variable						
	AGE->PW	0.0295	0.053	0.546	0.584	−0.076	0.1355
	Ethnicity->PW	−0.1202	0.034	−3.52	0.005	−0.187	−0.053
	Direct effect						
H1	FAE->PW	0.3819	0.056	6.812	0.0000	0.2716	0.4921
	Indirect moderating effect						
H2	FAE*CR->PW	0.3512	0.064	5.422	0.0000	0.2243	0.4796
H3	FAE*SE->PW	0.4010	0.056	6.916	0.0000	0.2798	0.5021

FAE, fine arts education; PW, psychological wellbeing; CR, creativity; SE, self-efficacy; LLC, lower limit confidence interval; ULCI, upper limit confidence interval.

## Discussion

The aim of this study was to find out the impact of fine arts education on psychological wellbeing among undergraduate students: moderating role of creativity and self-efficacy. Art fosters individuals in a multifaceted manner; it is also crucial for socialization or the process of integrating an individual into the society to which they belong. In a study, Sahin and Bagc (2012) stated that through art, individuals may easily communicate, acquire the ability to think critically, internalize the values in works of art and accumulate values, and develop into persons who contribute to social progress. Through art education, individuals develop artistic sensibility, demonstrate respect for differing viewpoints, safeguard their own culture, and accord other cultures the necessary importance. Additionally, art education serves as a tool for young people and adults to capture those experiences and perceptions that are significant for human development and one's engagement with the world in general. Through art education, individuals become more self-aware, acquire moral principles, and get a broader view on various subjects (Yeniasır and Gökbulut, 2018).

Following were the hypotheses of this study. First, fine arts education is positively associated with psychological wellbeing. The study's findings were consistent with the hypotheses. Previous research has found that visiting art museums reduces stress, which may promote health and wellbeing. The elements of the museum setting that facilitate treatment goals, including psychological, social, and environmental aspects, have been studied. Empirical evidence suggests that art improves people's health and wellbeing (Mastandrea et al., 2019). Similarly, art-based pedagogy is concerned with integrating an art form (e.g., theater, visual art-painting, music) with another subject matter in order to improve learning processes (Rieger and Chernomas, 2013). Moreover, previous research found that students could create creative tools, artwork, or campaigns that reflect how artists promote healthy work lifestyles and manage mental health issues (Siddins, 2021).

Secondly, creativity plays a moderating role between fine arts education and psychological wellbeing. The findings of this study were in line with previous literature. Prior research revealed that art education contributes to a model of education that offers individuals possibilities for free expression, integrates physical, intellectual, and creative talents, and facilitates dynamic and successful linkages between education, culture, and the arts. Art disciplines and practices are crucial to the intellectual, scholastic, cultural, social, and personal growth of adolescents. Therefore, art and art education are essential components of fundamental education (Romanescu and Özlem, 2020). Likewise, art education is crucial to the fulfilling of such demands since it permits the development of flexible and diverse thinking patterns, helps emotional development, and promotes learning and skill acquisition through cooperation (Romanescu and Özlem, 2020). Through imagination and creativity, we discover our identity and our source of healing. The more we comprehend the connection between creative expression and healing, the more we will recognize the healing power of the arts (Stuckey and Nobel, 2010). Participation in art education may not directly result in greater achievement; however, art education improves students' mental health, self-confidence, and life skills in several crucial ways. Additionally, art education fosters and sustains the innate creativity of young people (Roege and Kim, 2013).

Thirdly, self-efficacy plays a moderating role between fine arts education and psychological wellbeing. The results were consistent with the hypotheses. According to previous research, arts activities can involve aesthetic engagement, imaginative participation, sensory activation, emotional evocation, and cognitive stimulation. Depending on the nature of the art activity, it may also incorporate social interaction, physical activity, engagement with health-related topics, and involvement with healthcare facilities (Fancourt, 2017). Each component of arts activities can elicit psychological, physiological, social, and behavioral reactions that are in turn directly connected to health consequences (Fancourt and Finn, 2019). Significant positive correlations were found between self-efficacy and characteristics of psychological wellbeing, including autonomy, environmental mastery, personal progress, positive relationships, meaning in life, and self-acceptance. All aspects of psychological wellbeing were discovered to be positively connected (Kausar and Ahmad, 2021).

## Conclusion

The aim of this study was to explore the impact of fine arts education on psychological wellbeing of higher education students through moderating role of creativity and self-efficacy. The findings of this study supported the hypothesis, and the results of the study were in accordance with prior research. Results revealed that fine arts education is positively associated with psychological wellbeing. The findings of this study also affirm that the creativity and self-efficacy positively and significantly moderate the relationship between fine arts education and psychological wellbeing. Fine arts education is an important tool for developing human resources and contributes to the development of skills that countries require to profit from their cultural wealth. In this regard, countries that wish to build robust and sustained creative sectors are increasingly realizing that these capital and resources are critical to raising the country's socioeconomic level. Moreover, education in the fine arts is a long-term investment in people, and therefore, fine arts education should be carefully planned and its objectives should be well-defined.

This research will raise awareness among parents, students, and teachers in the future. As a result, parents and teachers in China's higher education sector have a better understanding of the value of fine arts education and how it affects students' psychological wellbeing. Similarly, creativity and self-efficacy play an important role in moderating the relationship between fine arts education and psychological wellbeing. Furthermore, the study's cross-sectional design is a limitation. In future studies, mixed methods will be used to investigate these variables in greater depth.

### Theoretical implications

This study makes an important contribution to the body of literature. This study was based on the significance of fine arts education on psychological wellbeing among undergraduate Chinese students and role of self-efficacy and creativity as a moderator. Previous literature supported the study hypothesis that fine arts education has a positive association with psychological wellbeing among Chinese students. Further, this study employs psychological wellbeing theory and theories of creativity to examine the conceptualized path.

### Practical implications

Following are the practical implications of this study. Students studying in this field should be made more aware of the importance of art education. More theoretical information should be provided in art schools, colleges, and universities, and students should be given more opportunities for practice in the field they are studying. Art courses should be added to the curricula of various stages under the Ministry of National Education, and students' sensitivity to art should be increased. Parents, particularly those without a higher education degree, should be advised to consider their children's opinions when making career choices. Expert educators/school guidance services should assist students in making career decisions.

The relationship between teaching and learning and how they work together is very important in traditional Chinese education. Teachers who are very good at what they do lay the groundwork for their students to learn and understand what they have learned easily. Teachers are the ones who teach their students what they know. They should not only tell people what to do, but also show them how to do it. Through what they do, they serve as an example and a guide. In addition to teaching, they will keep an attitude of active learning, improve their skills, and learn more. Teachers should be encouraged and helped to teach and do research by the higher education system. The goal of the study is to develop technical, practical, and theoretical abilities at the intersection of fine and decorative arts theory and practice. Furthermore, the directions and principles of artistic pedagogy and art therapy are followed in order to meet the new needs and take advantage of new opportunities as the creative and educational fields change in today's society.

### Limitations and future recommendations

Following are the limitations of the study. To ensure the generalizability of the results, it will be necessary to conduct additional study with a diverse population in the future. Due to the inclusion of a self-report questionnaire in the study, the sample could be skewed if students responded in a manner that made them appear socially acceptable. Prior research on study variables was limited. For future studies, qualitative research will be conducted on fine arts students through detailed interviews. A cross-cultural study should be conducted to investigate the norms influencing the psychological wellbeing of performing arts students. Moreover, future researchers should also study different moderators with fine arts education and psychological wellbeing for further relationship of these variables.

## Data availability statement

The raw data supporting the conclusions of this article will be made available by the authors, without undue reservation.

## Ethics statement

Ethical review and approval was not required for the study on human participants in accordance with the local legislation and institutional requirements. Written informed consent from the patients/participants or patients/participants legal guardian/next of kin was not required to participate in this study in accordance with the national legislation and the institutional requirements.

## Author contributions

Both authors listed have made a substantial, direct, and intellectual contribution to the work and approved it for publication.

## Conflict of interest

The authors declare that the research was conducted in the absence of any commercial or financial relationships that could be construed as a potential conflict of interest.

## Publisher's note

All claims expressed in this article are solely those of the authors and do not necessarily represent those of their affiliated organizations, or those of the publisher, the editors and the reviewers. Any product that may be evaluated in this article, or claim that may be made by its manufacturer, is not guaranteed or endorsed by the publisher.
